# Short structured general mental health in service training programme in Kenya improves patient health and social outcomes but not detection of mental health problems - a pragmatic cluster randomised controlled trial

**DOI:** 10.1186/1752-4458-7-25

**Published:** 2013-11-05

**Authors:** Rachel Jenkins, Caleb Othieno, Stephen Okeyo, Dan Kaseje, Julyan Aruwa, Henry Oyugi, Paul Bassett, Felix Kauye

**Affiliations:** 1 WHO Collaborating Centre, Institute of Psychiatry, PO 35, King’s College, De Crespigny Park, London, UK; 2 Department of Psychiatry, University of Nairobi, Nairobi, Kenya; 3 Great Lakes University, Kisumu, Kenya; 4 Statsconsultancy Ltd, London, UK; 5 University of Malawi, Zomba, Malawi

**Keywords:** Training programme, Mental health, Primary care, Kenya, Pragmatic cluster randomised controlled trial

## Abstract

**Trial design:**

A pragmatic cluster randomised controlled trial.

**Methods:**

*Participants:* Clusters were primary health care clinics on the Ministry of Health list. Clients were eligible if they were aged 18 and over. *Interventions:* Two members of staff from each intervention clinic received the training programme. Clients in both intervention and control clinics subsequently received normal routine care from their health workers. *Objective:* To examine the impact of a mental health inservice training on routine detection of mental disorder in the clinics and on client outcomes. *Outcomes:* The primary outcome was the rate of accurate routine clinic detection of mental disorder and the secondary outcome was client recovery over a twelve week follow up period. *Randomisation:* clinics were randomised to intervention and control groups using a table of random numbers. *Blinding:* researchers and clients were blind to group assignment.

**Results:**

*Numbers randomised:* 49 and 50 clinics were assigned to intervention and control groups respectively. 12 GHQ positive clients per clinic were identified for follow up. *Numbers analysed:* 468 and 478 clients were followed up for three months in intervention and control groups respectively. *Outcome:* At twelve weeks after training of the intervention group, the rate of accurate routine clinic detection of mental disorder was greater than 0 in 5% versus 0% of the intervention and control groups respectively, in both the intention to treat analysis (p = 0.50) and the per protocol analysis (p =0.50). Standardised effect sizes for client improvement were 0.34 (95% CI = (0.01,0.68)) for the General Health Questionnaire, 0.39 ((95% CI = (0.22, 0.61)) for the EQ and 0.49 (95% CI = (0.11,0.87)) for WHODAS (using ITT analysis); and 0.43 (95% CI = (0.09,0.76)) for the GHQ, 0.44 (95% CI = (0.22,0.65)) for the EQ and 0.58 (95% CI = (0.18,0.97)) for WHODAS (using per protocol analysis). *Harms:* None identified*.*

**Conclusion:**

The training programme did not result in significantly improved recorded diagnostic rates of mental disorders in the routine clinic consultation register, but did have significant effects on patient outcomes in routine clinical practice.

**Trial registration:**

International Standard Randomised Controlled Trial Number Register ISRCTN53515024.

## Background

Mental disorders are common in primary care across the world [1]. Specific interventions for single disorders or single client groups in low-income countries have been evaluated [2,3] but it remains challenging in Africa to scale up for all the mental disorders and client groups which commonly present to primary care, within existing human and financial resources, because on average only 0.7% of the health budget is spent on mental health services [4] with national health budgets often only around 10 USD per capita per year [5].

General integration of specialist programmes into general health systems may achieve better outcomes than more targeted disorder specific integration [6]. A few evaluations of such general rather than disorder specific integration have previously been conducted [7-10], and two randomised controlled trials in Iraq [11] and Malawi [12] also evaluated a general integration model, adapting the Kenya Medical Training College (KMTC) mental health primary care training toolkit described below. The Iraq trial was designed to examine the impact of the training on physician consultation skills as appraised by observations by research psychiatrists and by exit interviews with clients. The Malawi trial was designed to examine the impact of the training on diagnostic practice. The trial reported here was therefore designed to examine the impact of the training on rate of accurate routine clinic detection of mental disorder and client health and social outcomes, and quality of life, within the Kenyan public primary health care system.

Kenya, like a number of sub Saharan African countries, has a complex layered primary care system [13], with the first level representing the community, the second level representing dispensaries, the third level health centres, and the fourth level district hospitals and their outpatient clinics. Reported prevalence rates of mental disorders range from 26% to 63% in district hospitals [14-16]; 20% [17] to 46% [18] in health centres; and 11% in the community [19].

The KMTC mental health continuing professional development training programme is a 40 hour training course for primary care, which was devised for Kenya in 2004, piloted and subsequently systematically rolled out across nearly half of Kenya’s primary care staff in collaboration with the Government of Kenya in 2005–2010 [20,21]. The training programme uses a sustainable general health system approach, in which the content of the training is closely aligned to the generic tasks of the health workers for child health, reproductive health, communicable and non-communicable diseases, as set out in the Kenya national health sector strategic plan; and the training delivery was integrated into the normal national training delivery system. The content of training was also informed by earlier qualitative and quantitative studies of attitudes to mental health in the general health system, traditional healers and the community [14-17,22-24].

The 40 hour continuing professional development course consists of 5 modules. The first module gives an overview of core mental health concepts (mental health and wellbeing, contributors to good mental health, mental illness, causes and consequences of mental illness, the links between mental and physical health, the contribution of mental illness to disability adjusted life years and the Global Burden of Disease, and the contribution of mental health to the Millennium Development Goals). The second module covers important basic skills (communication skills, identifying clients with mental health problems, mental state examination, diagnosis, differential diagnosis, case formulation, care planning, giving psychosocial support, engagement with difficult clients, breaking bad news, community based rehabilitation, medication management, management of violence, domestic violence and child abuse). The third module covers the presenting symptoms, assessment, diagnosis and management of common neurological problems seen in primary care (epilepsy, dementia, delirium, Parkinson’s, headache). Similarly, the fourth module covers the presenting symptoms, multiaxial biopsychosocial assessment, diagnosis and management of mental disorders commonly encountered in primary care (depression, somatisation, dissociation, anxiety, panic, phobias, adjustment disorder, PTSD, sleep and eating disorders, male and female sexual disorders, alcohol abuse, drug abuse, acute psychosis, bipolar disorder, schizophrenia, childhood emotional and conduct disorders, ADHD, dyslexia, Autism Spectrum Disorder, learning disabilities and child abuse); Lastly, the fifth module covers the wider contextual issues of the Kenya Health Sector Strategic Plan, the Kenya Mental Health Policy, the Kenya policy on reproductive health, child health, malaria and HIV and the contribution of mental health to these health objectives; the Kenya mental health legislation and human rights; mental health promotion and prevention; potential roles of community health volunteers, and mechanisms for their training and supervision; traditional health practitioners, their advantages and disadvantages, and potential mechanisms for dialogue and liaison; health management information systems; roles and responsibilities at each level of the health system and local planning; disaster management; and consideration of how the learning from the course can be implemented within the participants’ clinics over the succeeding year.

Modules 3 and 4, which cover neurological and psychiatric disorders respectively, were specifically adapted from the WHO primary care guidelines [25,26], and follow the general structure of presenting symptoms, criteria for diagnosis, differential diagnosis, investigations, advice and information to the patient, advice and information to the family, psychosocial support, use of medicines if indicated and if available, criteria for referral, and community resources. They were tailored for Kenya in 2005 by an initial small group of psychiatrists, primary care staff and a leader of a mental health non-governmental organisation. This first Kenyan draft was circulated for consultation to professionals from primary care, psychiatry, the ministry of health, the University of Nairobi, and the professional and regulatory bodies for nurses and clinical Officers, and NGOs, who submitted comments which were used to create the second revision. The sections on child health, reproductive health, HIV and malaria were agreed with the relevant Ministry of Health (MOH) policy teams. Representatives from the National Council of Clinical Officers, the Nursing Council of Kenya and the MOH Divisions of Reproductive Health, Child and Adolescent Health and Malaria attended the 3 initial courses for trainers, and contributed cultural and specific health related revisions to the course and to the third and final draft of the guidelines and the teaching materials. The final guidelines [27] are endorsed by the Division of Mental Health, MOH, Kenya Medical Training College, Kenya Clinical Officer Council, Nursing Council of Kenya, Kenya Psychiatric Association, Department of Psychiatry, University of Nairobi, and the Kenya Schizophrenia Fellowship, which until now is the main indigenous mental health NGO in Kenya. The guidelines were distributed to all participants of the training programme, together with all slide handouts, and a copy of the Kenya Mental Health Act 1989. The overall course is approved by the Nursing Council of Kenya and the Kenya Clinical Officer Council for 40 hours credit for Continuing Professional Development of staff, recently mandatory in Kenya.

All five modules in the course are generally structured into several 30 minute and a few 60 minute topic based sessions, each of which is composed of roughly one third theory, one third queries and discussion, and one third taking part in a role play in which specific skills are rehearsed, followed by a demonstration role play which is then discussed by the group as a whole. Over the 40 hours course, all participants have to complete all 27 role plays.

Clinical skills rehearsed during the role plays include generic communication skills, giving psychosocial support, breaking bad news, training community health workers, biopsychosocial assessment and management of each mental disorder, assessment and management of suicidal risk, prescription of medicines, explanation of side effects and their management, and consideration of human rights and other ethical issues within the primary care clinical setting. World Psychiatric Association videos demonstrating specific skills are also shown.

The trainers who rolled out the training across Kenya were KMTC lecturers whose role is to deliver basic and post-basic health courses to nurses, clinical officers, pharmacists, physiotherapists and occupational therapists. They are themselves former nurses and clinical officers recruited from the health service to the training college, and have usually been given additional professional development by KMTC in how to train health workers.

Qualitative testing of the training programme included (i) iterative improvement of the course over the five years (ii) detailed collated written feedback from participants over the five years (iii) pre- and post-test evaluation of the first 1000 trained, showing change in mean score from 42% to 77% (p < 0.001 level) on a knowledge and attitude questionnaire covering the content of the different modules of the course, and (iv) Ministry of Health monitoring and evaluation of trained practitioners in several districts [20,21].

This paper therefore reports an exploratory trial, as a pragmatic cluster RCT, designed to test the effect of a low-cost training intervention, integrated with the national health sector reforms, (i) on the rate of accurate routine clinic detection of mental disorder, measured and analysed at the cluster level, (ii) on recovery (improved health and social outcomes and quality of life) of clients, measured at the individual participant level, and analysed allowing for the clustered nature of the data. The cluster design was chosen to minimise the risk of contamination between the practice of health workers in clinics were staff received the inservice training and clinics where staff did not receive the inservice training.

## Methods

### Study area

The study was conducted in Nyanza province, Kenya, as this was the region where the national training programme 2005/10 had hitherto trained fewest staff, and thus most clinics were eligible for study. The districts of Siaya, Bondo and Rachuonya were selected, all located around Kisumu near Lake Victoria. Livelihoods are based on subsistence farming, an extensive fishing industry along the lake, and some commercial business. The majority tribe is Luo. The area was the site of significant election violence in January 2007. Primary health care is delivered through level 2 dispensaries and level 3 health centres, with catchment populations of approximately 10 0000 and 30–50 000 respectively. Each health facility is staffed by one or more nurses and clinical officers on Ministry of Health salaries, and around 15–20 community health workers who are not remunerated by the Ministry of Health, but are now expected to receive small remuneration from the community.

### The study design

The study design is an exploratory pragmatic cluster RCT to compare practitioner and client outcomes of a trained group of health centres compared with an untrained group, using a post test design because of resource constraints. The cluster was the primary health care clinic (PHC). The study was designed to be as realistic an evaluation as possible of the impact of the training programme as delivered by routine trainers, and as implemented by routine health workers in routine practice. The study was therefore conducted in a field setting, using local trainers, and with local health workers conducting routine practice (see below for further details). We did not influence the local availability of medicines, or the health management information system. The project did supply good practice guidelines and handouts to those who attended the training course, and the project also provided a training course on mental health for the local district public health nurses whose role is to provide support and supervision to primary care.

Randomisation was conducted at the cluster level, namely PHC level rather than individual health worker level. If randomisation had taken place at individual health worker level, the risk of contamination between the practice of trained and untrained staff would be high, since they work closely in small teams [28].

The sample framework was the Ministry of Health list of all publicly funded primary care facilities in Siaya, Bondo and Rachuonya districts in Nyanza province. All public level 2 and 3 health facilities were eligible for randomisation, which was done by DK and the Great Lakes University Knowledge Management and Research Department, using a table of random numbers [29]. Level 2 and Level 3 clinics are distinguished by size and number of health workers. Generally level 2 clinics have 1 or 2 health workers, level 3 clinics have more. (Under the new Kenyan constitution, the distinction between level 2 and level 3 clinics is about to be lost, and all level 2 and level 3 facilities will be grouped into one level of health facility. Under the system extant at the time of the study, level 2 clinics did not refer patients to level 3, but rather directly to level 4 district hospitals). Centres where staff had previously received training from the KMTC mental health training programme were excluded from the study.

A random sample of 99 centres were selected by DK, stratified by health facility level, which were then randomly allocated by DK to intervention and control groups, resulting in 33 dispensaries and 16 health centres in the intervention group and 37 dispensaries and 13 health centres in the control group (29).

Within each clinic, 12 clients were selected for assessment and three month follow up (see procedures below). JA enrolled the clinics. The research assistants recruited the individual participants.

### Outcome measures

The primary outcome measure is the rate of accurate routine clinic detection of mental disorder, (defined as a comparison between the GHQ status of the client and the diagnosis recorded by the health worker in the routine clinic consultation register), and is measured at the cluster level.

The General Health Questionnaire is a screening instrument for psychological distress, reflecting a broad dimension of anxiety and depressive disorders, designed for use in primary care, and is available in several versions [30-32]. It has been shown to be a valid instrument for detection of psychiatric morbidity in both general medical settings and the community [30,33-35]. Originally a 60 item questionnaire, it has been variously abbreviated to 30, 28, 20 and 12 item versions. There are many validity studies of the 28 item, 30 item and 60 item versions including in Kenya and other developing countries [14,34,36-44] but relatively few of the 12 item version [14,36,42-46], although it has been validated in several African studies [47,48]. The GHQ-12 takes two minutes to complete by a literate person, and is very useful in situations where because of widespread illiteracy, the questions have to be read out to the respondent [47]. Studies have shown that the shorter versions are just as good if not better than the longer versions at identifying psychological distress [42]. The number of items with a morbid rating (using the GHQ scoring method) is counted to give a total GHQ score, and correlates highly with the total score derived in detailed standardised clinical assessments and other self-completion questionnaires. GHQ-12 rate of accurate identification of mental disorder is obtained by comparing GHQ-12 status of the patient with diagnoses made by the primary care professional (blind to the GHQ score) in the routine primary care records [49].

Patients with a GHQ score of 3 or more were deemed GHQ positive. The routine consultation register was checked by a research assistant to identify the diagnoses given by the primary care worker to each of the GHQ positive patients who were identified in each clinic. The identification index is the proportion of the GHQ positive clients in each clinic for whom a psychiatric diagnosis had been recorded in the routine clinic consultation register by the health worker.

For secondary outcomes we measured improved client outcomes in health (GHQ), disability (WHODAS) and quality of life (EQ-5D), and is measured at the individual participant level.

The WHO DAS II is a measure of disability that aims to reflect key features of the International Classification of Functioning (ICF) and to assess activity limitations and participation restrictions irrespective of diagnosis. The primary version of WHO DAS II is a fully structured 36-item lay interviewer administered instrument [50]. The ICF is structured around the broad components of Body functions and structure, activities (related to tasks and actions by an individual) and participation (involvement in a life situation), and additional information on severity and environmental factors. The WHODAS domains are mapped directly onto ICF’s Activity and Participation component. http://www.who.int/classifications/icf/en/.

EUROQOL-5D (EQ-5D): a standardised self-completed instrument for use as a measure of health outcome, applicable to a wide range of health conditions and treatments. It provides a simple descriptive profile and a single index value for health status and is recommended for use in cost-effectiveness analyses by the Washington Panel in Cost Effectiveness in Health and Medicine [51]. The EQ is scored by entering the score for each of five questions into a special validated calculator (where population norms are embedded in the calculation) which supplies a value ranging from 0 to 1, where 1 is very good quality of life and 0 is very poor. Thus an increase in EQ score indicates improvement, whereas decreases in GHQ score and in WHO DAS indicate improvement. Population norms have been calculated for a number of countries around the world, but Zimbabwe is the only African country with population norms. Therefore the norms for Zimbabwe were embedded in the special validated calculator for the EQ used in this study [52].

### Procedures for intervention and control groups

Two staff from each of the 49 centres in the intervention group were invited by JA, through the Provincial Medical Officer for Nyanza and the respective District Medical Officers, to attend the mental health training course. None were invited from the control group. Thus the intervention pertains to the cluster level. Five such courses trained 98 staff in total during May and June 2010. There were no drop outs. Staff in both intervention and control groups were free, as usual, to attend any other training course offered by the Ministry of Health or other agencies throughout the duration of the project. During the period of the project training courses on malaria, vaccinations and breast feeding were conducted in Nyanza.

The local KMTC trainers, who delivered the 5 courses for the intervention group of the RCT, had previously been trained as trainers for the course by RJ in 2005, received a one week refresher course by RJ in 2009, and had been delivering several training courses per year to front line health workers across Kenya in the intervening periods. Thus this RCT was a realistic evaluation of the impact of training by real service trainers in a field setting, without any immediately preceding added strengthening by master trainers, because such additional strengthening just prior to the research evaluation would not have been consistent with the normal resource constraints and practicalities of the Ministry of Health and the health service. The RCT was also a realistic evaluation of the impact of training on practice in that patients in both intervention and the control groups were treated as the health worker routinely decided, based on their own knowledge, experience and training.

For the primary outcome, all attenders in each clinic on their respective assessment day (around 3 months after training of the health workers) were assessed with the GHQ-12 and all diagnoses recorded by the health workers present in the clinic on that day were extracted from the clinic register. The primary outcome pertains to the cluster level. For the secondary outcomes, the first 12 consecutive GHQ positive clinic attenders (clients) were recruited by the research assistants and assessed with WHODAS and EQ on that same day; and hen reassessed 12 weeks later (November-December 2010) to examine improvement after 12 weeks on GHQ score, WHODAS and EQ 5D. The secondary outcome pertains to the individual level, but was analysed allowing for the clustered nature of the data.

The instruments were available in English and Kiswahili. They were administered to participants to self-complete as well as verbally by research workers in English or Kiswahili to those who could not read. Literacy rates in Kenya are over 90% for men and 80% for women [53]. To reduce the possibility of attrition bias [54], we paid the 12 participants per cluster £2 per day to complete their initial assessment day (3 months after training of the health workers) and follow up day 12 weeks later, as compensation for their transport costs and time. There were no changes to trial outcomes after the trial commenced.

### Inclusion criteria

Clinic criteria for entry were that they were on the Ministry of Health list of PHCs, were publicly funded, and had not previously been exposed to the KMTC mental health training course. Patient Criteria for entry were that they were over 18, were able to speak the language spoken by the researchers, did not have dementia or learning disability of such severity as to be unable to complete the questionnaires, and did not refuse to cooperate.

### Blinding

Allocation concealment was at the cluster level. The research assistants were blind to whether the clinic staff had received the mental health training course, and to whether patients were attending clinics with trained or untrained staff. JA, who organised the research assistants in the field, was not blind to the clinic status. The clinic staff were not blind as to whether they had received the training. Recruited clinic patients were not informed by the research assistants of whether their health workers had received the training, but we did not run a quantitative check on whether recruited clinic patients became aware of the trained status of their health workers over the course of the study.

### Calculation of sample size

The primary outcome is the detection rate of mental disorder in the centres (agreement/disagreement of staff diagnosis of any kind of mental disorder recorded in the routine clinic consultation register with patient rated GHQ score above the cut-off threshold) comparing the intervention and control centres. This outcome was measured at the centre level, rather than the patient level. In African countries the detection rate is expected to be 5%, and it was hoped that the intervention would increase this to 20%. The standard deviation of the detection rate was assumed to be 24% [55]. With a 5% significance level and 80% power it is calculated that 41 centres per group would be required for the study, a total of 82 centres. To allow for a centre level attrition rate of approximately 15-20%, the study aimed to recruit 100 centres into the study but the statistician ended up randomising only 99 centres because one of the names entered in the program used to do the randomisation was that of a regional area rather than a health facility itself.

The number of patients per centre was based on showing a difference between the main patient level outcomes, namely GHQ, EQ and WHODAS scores. A difference of 0.30 standard deviations between the groups for these outcomes was considered to be a clinically important difference. With a 5% significance level and 80% power, an individually randomised trial would require a total of 350 participants to detect this level of difference between groups.

Due to the cluster randomised design of the study, patient outcomes will not all be independent of each other, and so the calculated sample size requires inflating upwards. We anticipated an intra-cluster correlation of 0.2, which was considered reasonably conservative for patient level quality of life outcomes, especially as they will not be related to each other. Assuming this ICC value, and that there are 100 centres, it is calculated that 10 patients per centre would be required to show a difference of 0.3 standard deviations between groups. To allow for a patient level attrition rate of approximately 15%, it was decided to follow-up 12 patients per practice.

### Statistical methods

We carried out two overall analyses, using Stata version 12 [56], firstly an Intention To Treat (ITT) analysis in which clinics were dealt with according to their initial randomisation (and so 4 control clinics were staff had erroneously received training were nonetheless treated as controls); and secondly a Per Protocol (PP) analysis, which excluded those control clinics where staff had erroneously been sent for training. The primary outcome was the detection rate of mental disorder in the centres. We had initially planned to use a Chi Square analysis but due to the unexpected finding of a large number of zero values, this was categorised as being zero or non-zero, and Fisher’s exact test was used to compare between study arms’, Differences in the secondary outcomes (GHQ, EQ and WHO DAS) were assessed at recruitment (initial assessment of clients), at follow-up of clients 12 weeks later, and in terms of the change from initial to follow-up assessments. To allow for the clustered nature of the data, the analysis was performed using multilevel linear regression. Two-level models were used with patients nested within clinics. The analysis of the changes in scores from initial assessment day to follow-up 12 weeks later was made using the change in scores as the outcome, and adjusting for baseline scores.

### Ethical considerations, explanation and consent

The study was registered and approved by the Kenya National Council for Science and Technology, Kenyatta National Hospital Ethics Committee, The Ethical Research Committee of Great Lakes University of Kisumu, and King’s College London ethics committee. Clients and health workers were approached for written informed consent, after randomisation. The trial is registered at International Standard Randomised Controlled Trial Number Register as ISRCTN53515024 and the full trial protocol can be accessed at http://www.controlled-trials.com/ISRCTN53515024/, Additional files 1 and 2 comprise the consort statement extension for pragmatic trials and for cluster randomized controlled trials respectively.

## Results

**Figure 1:**
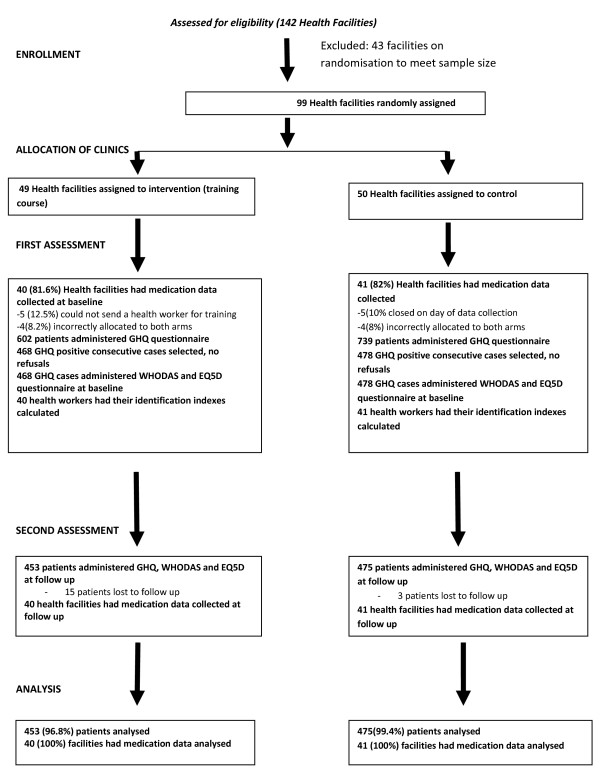
Flow chart of trial.

Figure 1 shows the trial profile. The flow diagram included all clinics which had data collected on the different outcomes. For simplicity, the flow diagram does not indicate that some clinics had data missing on identification index while others had data missing on several other variables. 41 intervention clinics and 36 control clinics had data for the calculation of identification index.

On the research day, there were 119 health workers in the study clinics who agreed to take part in the study: 55 (46.22%) in the control arm (30 (54.55%) female and 25 (44.45%) male, 8 (14.55%) clinical officers and 47 (85.45%) nurses) and 64 (53.78%) in the intervention arm (35 (54.69%) female and 29 (45.31%) male, 12 (18.75%) clinical officers and 52 (81.25%) nurses. The clinics and clients were each first assessed in July, August, September 2010 and followed up 12 weeks later in October, November and December 2010. The trial ended when all assessments were complete.

Table 1 shows the clinic response rates, and the presence of baseline medications in intervention and control clinics. It can be noted that medications were generally scarce in the clinics, with more than half having no anti psychotics or antidepressants at the time of the study. The only significant difference between intervention and control groups was that the intervention group had a higher proportion of clinics with benzodiazepines than control clinics. In Table 1, clinic factors are only reported for 41 intervention and 40 control clinics respectively, as the remaining clinics had missing data on these variables at baseline.

**Table 1:** Characteristics of clinics

**Variable**	**Control**	**Intervention**	**Difference (95% CI)**	**P-value**
**Clinic factors**	**n = 41**	**n = 40**		
Clinic attendance > 100/wk ^(*)^	24 (65%)	17 (45%)	0.17 (0.16, 1.10)	0.08
Antipsychotic present ^(†) (*)^	7 (18%)	3 (8%)	0.37 (0.10, 1.44)	0.16
Antidepressant present ^(†) (*)^	6 (15%)	4 (10%)	0.61 (0.17, 2.22)	0.47
Benzodiazepine present ^(†) (*)^	10 (27%)	21 (54%)	3.15 (1.22, 8.13)	0.02
Antiepileptic present ^(†) (*)^	16 (42%)	12 (30%)	0.59 (0.23, 1.48)	0.27

(†) Present defined as drugs fully present.

(*) Number (%) presented for each group. Differences between groups presented as odds ratio.

Table 2 shows the sociodemographic characteristics of the selected 12 patients per clinic. The only significant difference between intervention and control groups was that the intervention group had a higher proportion of people who were single, divorced or widowed.

**Table 2:** Sociodemographic characteristics of selected group of 12 patients per clinic

**Variable**	**Control**	**Intervention**	**Difference (95% CI)**	**P-value**
**Patient factors**	**n = 475**	**n = 453**		
Female ^(*)^	361 (76%)	329 (73%)	0.81 (0.52, 1.24)	0.36
Age (continuous) ^(**)^	32.8 (13.2)	34.6 (14.5)	1.7 (−0.6, 4.0)	0.16
Age 36+ ^(*)^	89 (38%)	242 (35%)	1.37 (0.99, 1.90)	0.06
Single/Divorced/Widowed ^(*)^	83 (18%)	122 (27%)	1.87 (1.22, 2.87)	0.004
Not Luo tribe ^(*)^	24 (5%)	14 (3%)	0.69 (0.14, 2.49)	0.64
4+ children ^(*)^	199 (42%)	191 (42%)	1.01 (0.72, 1.43)	0.95
Left education aged 17+ ^(*)^	201 (44%)	172 (40%)	0.88 (0.61, 1.26)	0.47

(*) Number (%) presented for each group. Differences between groups presented as odds ratio.

(**) Mean (SD) presented for each group. Mean difference between groups also reported.

Table 3 shows the rate of routine detection of mental disorder in the clinics, 3 months after staff from the intervention clinics had received training, showing that at twelve weeks after training of staff from the intervention group, the rate of routine detection of mental disorder was greater than 0 in 5% of the intervention clinics versus 0% of the control clinics in both the intention to treat analysis (p = 0.50) and the per protocol analysis (p =0.50).

**Table 3:** GHQ rate of accurate identification of mental disorder by health workers in the clinics

**Analysis type**	**Identification index**	**Control N (%)**	**Intervention N (%)**	**P-value**
Intention to treat analysis	Zero non-zero	36 (100%) 0 (0%)	38 (95%) 2 (5%)	0.50
Per protocol analysis	Zero non-zero	33 (100%) 0 (0%)	38 (95%) 2 (5%)	0.50

Of the 972 patients assessed at baseline, 928 patients completed the 12 week follow up, giving a follow up rate of 95.5%. Table 4 shows the mean GHQ, EQ and WHO DAS scores at recruitment (initial assessment day) and follow up (12 weeks later) in clients attending the control and trained clinics, and the mean change in scores over that time adjusted for baseline score. The change in scores over the 12 week period between first and follow-up assessment of clients was significantly greater in the clients in the intervention than in the control group in both ITT and PP analyses. Indeed, none of the three outcomes varied significantly between first assessment and follow-up in the control group, while in the intervention group there was a significant improvement in all three outcome measures over time, both when assessing difference in raw scores and after adjustment for first assessment baseline, in both ITT and PP analyses.

**Table 4:** Recovery of clients

**Outcome**	**Phase**	**Control**		**Intervention**		**Difference**	**P value**
		**N**	**Mean (SD)**	**N**	**Mean (SD)**	**Mean (95% CI)**	
**Intention to treat analyses**							
GHQ	Baseline	463	5.80 (2.37)	447	6.73 (2.38)	0.92 (0.24, 1.60)	**0.008**
	Follow-up	463	5.61 (2.58)	447	5.33 (2.66)	−0.30 (−1.08, 0.48)	0.45
	Change ^(*)^	463	−0.48	447	−1.11	−0.63 (−1.27, 0.01)	0.05
EQ	Baseline	447	0.66 (0.19)	438	0.63 (0.20)	−0.04 (−0.08, 0.01)	0.09
	Follow-up	447	0.66 (0.25)	438	0.72 (0.19)	0.06 (0.01, 0.11)	**0.02**
	Change ^(*)^	447	0.01	438	0.09	0.07 (0.04, 0.11)	**<0.001**
WHODAS	Baseline	462	25.6 (8.9)	437	29.8 (8.7)	4.0 (1.3, 6.7)	**0.003**
	Follow-up	462	26.8 (11.6)	437	25.5 (9.2)	−1.6 (−5.1, 1.8)	0.36
	Change ^(*)^	462	0.1	437	−3.1	−3.3 (−5.8, −0.7)	**0.01**
**Per protocol analyses**							
GHQ	Baseline	418	5.80 (2.37)	447	6.73 (2.38)	0.91 (0.23, 1.60)	**0.009**
	Follow-up	418	5.79 (2.54)	447	5.33 (2.66)	−0.48 (−1.26, 0.29)	0.22
	Change ^(*)^	418	−0.32	447	−1.12	−0.81 (−1.45, −0.17)	**0.01**
EQ	Baseline	398	0.65 (0.19)	438	0.63 (0.20)	−0.02 (−0.07, 0.02)	0.26
	Follow-up	398	0.65 (0.25)	438	0.72 (0.19)	0.07 (0.02, 0.13)	**0.006**
	Change ^(*)^	398	0.01	438	0.09	0.08 (0.04, 0.12)	**<0.001**
WHODAS	Baseline	418	25.7 (8.9)	437	29.8 (8.7)	3.9 (1.2, 6.7)	**0.005**
	Follow up	418	27.3 (11.8)	437	25.5 (9.2)	−2.2 (−5.7, 1.4)	0.23
	Change ^(*)^	418	0.6	437	−3.2	−3.8 (−6.4, -1.2)	**0.004**

(*) Assessed by analysing change in scores, adjusting for baseline scores. Marginal means presented. p values in bold all significant at <0.05, or greater (see text).

Using change scores adjusted for baseline, over the succeeding 3 months, in the ITT analysis, the clients in the trained health facilities improved by 1.11 units on the GHQ, 0.09 units on EQ and 3.1 units on WHODAS compared to 0.48, 0.01 and 0.1 respectively in the untrained health facilities, resulting in mean differences of 0.63 units on the GHQ, 0.07 on the EQ and 3.3. units on WHODAS with p values significant at < 0.05, <0.001 and <0.01 respectively. The standardised effect sizes were 0.34 (95% CI = (0.01,0.68)) for the GHQ, 0.39 (95% CI = (0.22,0.61)) for the EQ and 0.49 (95% CI = (0.11,0.87)) for WHODAS in the ITT analysis.

In the PP analysis, the clients in the trained health facilities improved by 1.1 units on the GHQ, 0.09 units on EQ and 3.2 units on WHODAS compared to 0.3, 0.0 and 0.6 respectively in the untrained health facilities, resulting in mean differences of 0.8, 0.08 and 3.8, with p values significant at <0.01, <0.001 and <0.004 respectively. The standardised effect sizes were 0.43 (95% CI = (0.09,0.76)) for the GHQ, 0.44 (95% CI = (0.22,0.65)) for the EQ and 0.58 (95% CI = (0.18,0.97)) for WHODAS in the PP analysis.

Table 5 shows the difference in numbers of days that patients were unable to perform activities. The groups were found to differ at baseline for the number of days that the subjects were unable to perform activities. This was significantly higher in the intervention group, with an average of 58% more days in this group. The groups were not significantly different in this outcome at follow-up. The results for the change over time suggested some evidence of greater reduction in the intervention group than in the control group. However, this difference was not quite statistically significant (p = 0.08). The number of days where clients were unable to perform activities at follow-up (after accounting for baseline differences) were around 20% lower in the intervention group.

**Table 5:** Days unable to perform activities in last month

**Outcome**	**Phase**	**Control mean (SD)**	**Intervention mean (SD)**	**Difference ratio (95% CI)**	**P value**
Unable to	Baseline	3.5 (6.0)	5.4 (6.5)	1.58 (1.24, 2.01)	**<0.001**
Perform	Follow-up	4.5 (7.8)	2.8 (5.0)	0.92 (0.73, 1.16)	0.49
activities	Change ^(*)^	0.9 (7.9)	−2.5 (7.0)	0.81 (0.65, 1.02)	0.08

(*) Ratio assessed by analysing follow-up values, adjusting for baseline values.

No harms were identified during the trial.

## Discussion

### Overview of findings

This study is a pragmatic cluster randomised controlled trial to determine the impact of a comprehensive, standardised and structured mental health training programme for primary care workers, developed for the KMTC national mental health CPD programme [13,20,21], on routine clinic detection of mental disorder and client outcomes over a twelve week period in a routine clinical setting. The improvement in primary outcome (GHQ identification index) was not significant, but there were significant improvements in secondary outcomes (improvements over a 12 week period in GHQ, WHODAS and EQ in clients attending the intervention clinics where two staff had been trained, relative to the clients attending control clinics where no staff had been trained) in both ITT analysis and the less conservative PP analysis. Thus despite the lack of impact on our primary outcome, nonetheless the intervention made a significant difference to patient wellbeing. The possibility of recruitment bias was reduced because the research assistants who recruited the clients had no knowledge of which group the clinics belonged to, and the possibility of attrition bias was reduced by compensating participants’ transport costs and time [54,55].

In relation to the primary outcome, the detection of mental disorders by the health workers was much lower than anticipated in both groups. We had assumed averages of 5% and 20% in the untrained groups and trained groups respectively in the power calculation. However, in the trial, it was 0% for most clinics. As a result of this low value the study lacks adequate power to show a difference in this outcome between groups. We would have needed around 190 clinics per arm to show a 5% difference based on the results (with 80% power). In relation to the secondary outcome, we had calculated adequate sample size, and the results have shown that the training programme, accompanied by good practice guidelines, has achieved a significant improvement in patient recovery in a real field setting, despite the poor supply of essential psychotropic medicines in a large proportion of the clinics, and despite the absence of systematic support and supervision about mental health from the district level.

### Potential reasons for improvement in client recovery despite lack of improvement in clinic recording of mental disorders, and despite the poor medicine supply

Although it appears surprising that there was significant improvement in secondary outcomes but not in the primary outcome, this may be partly due to two key factors. Firstly, the primary outcome was based on the health worker diagnosis in the clinic consultation register. This register contains one line per consultation, for name, age, sex and diagnosis. Thus the register does not lend itself to multiaxial diagnoses, and the health workers have hitherto been oriented by their basic training and previous clinical practice to prioritise physical illness above mental illness. During our study therefore it is likely that the presence of a physical disorder would routinely get preferentially recorded in the consultation register at the expense of a co-existing disorder such as depression. So it may not be so much a lack of recognition of a psychiatric diagnosis but rather that the psychiatric diagnosis is overshadowed by a physical diagnosis. More complex multiaxial health management information systems would have enabled health workers to record mental disorders even in the presence of presumed physical illness.

Secondly, the training delivered for this study emphasised a multiaxial biopsychosocial approach to assessment, diagnosis, and management of all clients including those with physical illness, and those attending routine reproductive health care, and so it is likely that such a perspective and approach, when implemented in routine practice, would lead to a greater focus by the health workers on the social aspects of causation and consequences of all illnesses, and hence better understanding of the illness, delivery of more focused psychosocial support to the client, and hence improved health and social outcomes.

Data collected during the trial demonstrated that the availability of medicines in the clinics had become worse than expected from experience in previous years, and dialogue with the Ministry of Health and donors indicated that this was a general problem across Kenya due to a combination of factors including the contemporaneous re-organisation of Kenya Medical Supplies Agency (KEMSA), and the shift from the push (national distribution of standard drug kits) to the pull system (local ordering by clinics according to need) of distribution of medicines, and government resource constraints. Due to the poverty levels in the districts studied [57] most people cannot afford to buy medications from private chemists which are available in the major towns. It is therefore noteworthy that the trial achieved significant client recovery despite the paucity of medicines, and suggests that it is the psychosocial aspects of the health worker-client interaction which were therapeutic.

### Advantages and limitations of the pragmatic design

This trial is a pragmatic trial [58,59] which aimed to be as realistic an assessment of impact of the training programme as possible, taking place in 2010 in a province distant from the capital city, with training delivered by local Kenyan trainers who had been trained in 2005, and received a refresher course in 2009; and in the prevailing context of limited medicine supply, district supervision and health management information systems. The outcome measures and follow up period were selected to inform ministry of health decision making about the value of investment in mental health inservice training for primary care staff. We only trained two staff per clinic, and were not able to afford to train all staff in the intervention clinics. On average, only half of all the health workers employed in the intervention clinics were trained by the programme. We could not ensure that clients in the intervention clinics were actually assessed and managed by staff who had been directly trained by the programme, nor do we have data on what proportion of clients in the intervention clinic were assessed and managed by staff who had been directly trained by the programme. The likelihood is that patients may see different members of staff at different consultations rather than consistently see the same health worker. This means that the measured effect of the training on diagnostic identification and on client outcomes was diluted by health workers in the intervention clinics who had not been trained. We consider these constraints to reflect the realistic practicalities of any ministry of health training programme routinely rolled out in the field, and consider therefore that the impact found in this trial reflects what may reasonably be expected from such a routine ministry of health training programme.

### Limitations of the post test design

Despite the randomisation process, there were significant differences between intervention and control clinics in baseline GHQ, WHODAS and EQ scores. Of course, even with randomization, significant differences can occur, and we expect that 5% of the comparisons will be significant at the .05 level. Another possible explanation is that this is a post –test design, where the recruitment and first assessments of clients were undertaken 3 months after the randomisation process and training of the health workers, so it may be that the health worker training had already had an impact on client behaviour such that people with mental health problems had become more likely to preferentially attend the trained clinics. In any event, our analyses adjusted for these baseline differences although this would not have corrected for unknown variables that may relate to outcome. This is a weakness of the post-test study design. However, if patients had had baseline measurements taken before randomisation in order to eliminate the possibility of selection bias, then a 3 month follow up would have to have been conducted immediately after training of the health workers which would have been before the health workers would have had time to assimilate the training and alter their practice in meaningful ways. Alternatively the follow up period could have been six months instead of three, but this would have run the risk of more clients lost to follow up.

### Comparison with other studies

Comparing this study with others in the literature is somewhat problematic as our study is of a complex comprehensive training intervention (a multicomponent training programme covering a wide variety of concepts, policy and practice issues, skills and disorders, and accompanied by good practice guidelines) for nurses and clinical officers, whereas other studies have tended to be about training general practitioners about one specific disorder, namely depression, often with one specific intervention such as Cognitive Behaviour Therapy.

A systematic review and meta-analysis of whether GP training in depression care affects patient outcome assessed 108 articles, 11 of which met the inclusion criteria, all in high income countries [60]. Of these 11 studies, education and training was the sole intervention in 3, while 4 used guidelines and guideline explanation as well. The 3 studies which solely provided physician training found no change in symptom severity, while the 4 studies which introduced guidelines and used them during practitioner training found a significant improvement in symptoms at mid and long term follow up. Thus training alone of GPs did not result in improved patient outcomes, but the addition of guidelines and the use of more complex interventions gave a significant reduction in depression symptomatology. Our KMTC training intervention also used good practice guidelines which were tightly aligned with the content of the teaching materials, and which emphasised the biopsychosocial approach to supporting clients and their families. The training programme was also relatively comprehensive, covering multiple disorders and interventions, as well as policy, service and practice issues.

Similar positive findings have been found in middle income countries. For example, integration of mental health in primary care has been implemented in rural areas of Iran since the 1980s, and a recent ten year prospective uncontrolled, community-based study was conducted on the 85000 urban and rural inhabitants of Chaharmahal and Bakhtyari province in Iran from 1999 to 2009 [61]. Interestingly, the study was not based in the front line health posts which were the original focus of integrated mental health care in Iran, but in the next tier of health centres staffed by general practitioners, who made diagnoses and referred to psychiatrists for confirmation of the diagnosis. This study also found that the diagnostic rate by GPs was relatively low compared to that found in western studies. In Chile, a randomised controlled trial of depressed women in 3 primary care centres found a significant impact of teaching stepped care, psychoeducation, structured systematic follow up and drug treatment for patients with severe depression [62].

Two trials of this particular KMTC training programme, with both training programme and accompanying guidelines adapted for the respective countries, have already been reported and have demonstrated significant improvements in physician interviewing and management styles in Iraq [11], and diagnostic practice in Malawi [12]. The Malawi study used the individual patient record card which was more conducive to recording multiaxial diagnoses than the clinic register.

Our study reported here is not the first study in Sub Saharan Africa to obtain positive outcomes for clients when staff are trained to recognize and manage common mental disorders, but it is the first to obtain positive outcomes through using the local training system and the local primary care implementers working as normal. Thus the trainers were KMTC personnel, working as part of the routine Ministry of Health system for delivering continuing professional development, the implementers were routine primary health care staff working as normal in their clinics. Evidence that outcomes can be influenced by such transfer of knowledge and skills is exciting from a sustainability perspective. The fact that the training programme also included commonly co-morbid infectious and non-infectious conditions and system level change approaches may be other aspects which assisted impact and which may assist sustainability.

In India and Pakistan [63], a naturalistic demonstration study argued that integrating mental health into primary care is likely to be cost effective. Chisholm et al. [64] international have since estimated the population level cost effectiveness of evidence based depression interventions and their contribution towards reducing the current burden of depression. They found that evaluated interventions have the potential to reduce the current burden of depression by 10-30%, and commented that current levels of burden can only be reduced significantly if there is substantial increase in treatment coverage. In low resource settings as well as in high income countries, such coverage can only be achieved by integration into primary care.

### Relevance of the course design

In general, we consider that the training course may have had the impact it did because its relatively comprehensive design and content, the overarching emphasis on the biopsychosocial approach, and its attention to wider practice organisational issues. The course does not just include specific knowledge transfer about assessment and management of one or two disorders, but instead includes most of the neurological and psychiatric disorders likely to be seen in primary care. The course is highly competency based, with all participants having to complete at least 27 observed role plays. The course does not just focus on mental and neurological disorders, but also contains modules on psychosocial skills, medication management, the links between mental health and physical health, especially child health, reproductive health, malaria and HIV; on the wider links between health, education, employment and criminal justice issues; on generic health policy, mental health policy, human rights, health management information systems, and linkages with community volunteers and traditional health practitioners. We consider the wider scope of the course may assist its subsequent practical implementation in the clinic, so that all patients attending the clinic receive a more biopsychosocial approach to assessment and management than they did prior to the staff training.

### Triangulation of study findings

We did not have the resources to conduct clinic observations during the 12 week follow up period to examine how well the health workers conducted assessment and management of patients, but we did conduct focus group studies with some of the health workers and clients from the intervention and control groups which are reported elsewhere [65-67], and which suggested that the health workers in the intervention group perceived an increase in their communication, diagnostic and counseling skills, and that the patients in the intervention group noticed and appreciated these enhanced skills, while health workers and clients in the control group were both aware of the lack of these skills.

### Lack of attention to mental health in other training courses

Nyanza is a province with a high prevalence of HIV - the highest rates in Kenya are recorded around the Lake Victoria beaches where it is linked to the fishing trade and commercial sex trade, and so the other non-mental health trainings that both control and intervention groups would be likely to have previously received include HIV training, as well the training on vaccinations, breast feeding, nutrition, and paediatrics which occurred during the study period. However, the amount of mental health promotion and mental disorder assessment and management covered in Kenyan HIV training programmes is minimal or non-existent. The focus of such HIV training is on pre-test and post-test counselling, and both control and intervention clinic staff would have been equally exposed to it, so this could not have interfered with the results of this study. The focus group discussions conducted with staff after the end of the RCT [65-67] also supported the view that such HIV trainings do not pay significant attention to mental health issues.

### Implications for research and training

The findings suggest the need for further research to identify the barriers to disorder identification after staff training, in these primary health care contexts. Implications for other training programmes include the likely value of including the provision of good practice guidelines synchronous with the training programme; of including contextual health policy, service and practice issues; of including comorbidity issues with infectious and non-infectious diseases, child health and reproductive health; and of increasing the focus on the importance of recording mental health data within the routine health management information system, and thus overall of taking a generic health system strengthening approach.

## Conclusions

A 40-hour comprehensive structured interactive mental health training programme accompanied by good practice guidelines, delivered by local Kenyan trainers in a real field setting (covering both sexes and all the adult age range), did not result in significantly improved recorded diagnostic rates of mental disorders in the clinics but did result in significant improvement in client recovery and in days unable to perform activities, despite the prevailing conditions of poor medicine supply and lack of district supervision. Such training is therefore worth further evaluation.

## Abbreviations

ITT: Intention to treat analysis; KMTC: Kenya medical training college; NGO: Non governmental organisation; PP: Per protocol analysis; RCT: Randomised controlled trial.

## Competing interests

RJ has received previous grants from DFID and Nuffield. SO, FK, HO, JA, PB, CO have no competing interests. The grant from DFID covers the processing charge of articles arising from this project.

## Authors’ contributions

RJ designed the study, gave overall supervision to the study, wrote the first draft of the manuscript, and coordinated all revisions. DK led the randomisation process. SO and CO gave local supervision to the study. JA recruited clinics and patients, and supervised the research assistants who collected the data. FK and PB conducted the main analyses; HO conducted some preliminary analyses. All authors contributed to later drafts of the manuscript. The corresponding author had full access to all the data in the study and had final responsibility for the decision to submit for publication. All authors read and approved the final manuscript.

## Authors’ information

RJ is a consultant psychiatrist, and worked for the UK Department of Health 1988–1996 as Principal Medical Officer of Health, responsible for mental health policy, and subsequently directed the WHO Collaborating Centre for Research and Training at the Institute of Psychiatry 1997–2012, working with a variety of low and middle income countries, and is now Professor Emeritas at Kings College London where she continues to work on international research, training and policy. She sits on England’s national suicide prevention strategy advisory committee. She has been a trustee of a number of national and international mental health NGOs, and is currently a trustee of Partnership for Children, and chair of the trustees of the Educational Trust for Health Improvement through Cognitive Studies.

## Supplementary Material

Additional file 1 The consort statements for pragmatic trials. Click here for file

Additional file 2 The consort statement for cluster randomised controlled trials. Click here for file

## References

[B1] Ustun TB Sartorius N Mental Illness in General Health Care: An International Study 1995 Chichester: Wiley

[B2] Rahman A Malik A Sikander S Roberts C Creed F Cognitive behaviour therapy-based intervention by community health workers for mothers with depression and their infants in rural Pakistan: a cluster-randomised controlled trial Lancet 2008 372 902 909 18790313 10.1016/S0140-6736(08)61400-2 PMC2603063

[B3] Patel V Weiss HA Chowdhary N Naik S Pednekar S Chatterjee S De Silva MJ Bhat B Araya R King M Effectiveness of an intervention led by lay health counsellors for depressive and anxiety disorders in primary care in Goa, India (MANAS): a cluster randomised controlled trial Lancet 2010 376 2086 2095 21159375 10.1016/S0140-6736(10)61508-5 PMC4964905

[B4] World Health Organisation Mental Health Atlas 2005 Geneva: World Health Organisation http://www.who.int/globalatlas/pgrms/mentalhealth

[B5] Zarocostas J African countries need to allocate more of their budgets to health, says WHO Brit Med J 2011 342 1280

[B6] Atun R de Jongh TE Secci FV Ohiri K Adeyi O Car J Integration of priority population, health and nutrition interventions into health systems: systematic review BMC Public Health 2011 11 780 21985434 10.1186/1471-2458-11-780 PMC3204262

[B7] Jenkins R Bobyleva Z Goldberg D Gask L Zacroeva A Potasheva A Krasnov A McDaid D Integrating mental health into primary care in Sverdlovsk Mental Health in Family Medicine 2009 6 1 29 36 22477885 PMC2777595

[B8] Zakroyeva A Goldberg D Gask L Leese M Training Russian family physicians in mental health skills Euro J Gen Prac 2008 14 19 22 10.1080/13814780701855690 18464168

[B9] Schulsinger F Jablensky A The national mental health programme in the United Republic of Tanzania. A report from WHO and DANIDA Acta Psychiatrica Scandinavica Supplement 1991 364 1 132 1867110

[B10] de Jong JT A comprehensive public mental health programme in Guinea-Bissau: a useful model for African, Asian and Latin-American countries Pychol Med 1996 26 97 108 10.1017/s0033291700033742 8643767

[B11] Sadik S Abdulrahman S Bradley M Jenkins R Integrating mental health into primary care in Iraq Mental Health Family Med 2011 8 39 49 PMC3134212 22479291

[B12] Kauye F Rahman A Jenkins R Training primary health workers in mental health and its impact on diagnoses of Common mental disorders in primary care of a developing country, Malawi: a cluster-randomized controlled trial Psychol Medavailable on CJO2013. doi:10.1017/S0033291713001141 10.1017/S0033291713001141 23721658

[B13] Kiima D Jenkins R Mental health policy in Kenya - an integrated approach to scaling up equitable care for poor populations Int J Mental Health Syst 2010 4 19 10.1186/1752-4458-4-19 PMC2907308 20584266

[B14] Dhadphale M Ellison RH Griffin L The frequency of psychiatric disorders among patients attending semi-urban and rural general out-patient clinics in Kenya Brit J Psychiatry 1983 142 379 383 6850176 10.1192/bjp.142.4.379

[B15] Dhadphale M Ellison RH The frequency of mental disorders in the out-patients of two Nyanza hospitals Central African J Med 1983 29 29 32 6850816

[B16] Dhadphale M Cooper G Cartwright-Taylor L Prevalence and presentation of depressive illness in a primary health care setting in Kenya Am J Psychiatry 1989 146 659 661 2785347 10.1176/ajp.146.5.659

[B17] Ndetei DM Muhangi J The prevalence and clinical presentation of psychiatric illness in a rural setting in Kenya Brit J Psychiatry 1979 135 269 272 486853 10.1192/bjp.135.3.269

[B18] Kiima D Psychiatric morbidity among patients attending a primary care health facility from a deprived community in Nairobi 1987 Nairobi: University of Nairobi

[B19] Jenkins R Njenga F Okonji M Kigamwa P Baraza M Ayuyo J Singleton N McManus M Kiima D Prevalence of CMD in a rural district of Kenya, and sociodemographic risk factors Int J Environmen Res Pub Health 2012 9 1810 1819 10.3390/ijerph9051810 PMC3386589 22754474

[B20] Jenkins R Kiima D Njenga F Okonji M Kingora J Kathuku D Lock S Integration of mental health into primary care in Kenya World Psychiatry 2010 9 118 120 20671901 10.1002/j.2051-5545.2010.tb00289.x PMC2911092

[B21] Jenkins R Kiima D Okonji M Njenga F Kingora J Lock S Integration of mental health in primary care and community health working in Kenya: context, rationale, coverage and sustainability Mental Health Fam Med 2010 7 37 47 PMC2925163 22477921

[B22] Sebit MB Prevalence of psychiatric disorders in general practice in Nairobi East African Med J 1996 73 631 633 8997839

[B23] Muga F Jenkins R Training, attitudes and practice of district health workers in Kenya Soc Psychiatr Psychiatric Epidemiol 2008 43 477 482 10.1007/s00127-008-0327-z 18327522

[B24] Muga F Jenkins R Public perceptions, explanatory models and service utilisation regarding mental illness and mental health care in Kenya Soc Psychiatr Psychiatric Epidemiol 2008 43 469 476 10.1007/s00127-008-0334-0 18427705

[B25] World Health Organisation Diagnostic and Management Guidelines for Mental Disorders in Primary Care:ICD-10 Chapter V Primary Care Version Goettingen, Germany: Hogrefe and Huber

[B26] World Health Organization Collaborating Centre WHO Guide to Mental and Neurological Health inPrimary Care (2e) 2004 London: Royal Society of Medicine

[B27] Mental Health in Primary Care Diagnostic and Treatment Guidelines Based on the WHO Primary Care Guidelines for Mental Disorders adapted for Kenya 1.5.06 2006 Kenya: Ministry of Health

[B28] Donner A Klar N Pitfalls of an controversies in cluster randomization trials Am J Pub Health 2004 94 416 422 14998805 10.2105/ajph.94.3.416 PMC1448267

[B29] McClure JT Dietrich FH Statistics 1994 Dellen: Macmillan College Publishing Co 909 911

[B30] Goldberg D The detection of Psychiatric illness by questionnaire Maudsley Monograph, vol. No. 21 1972 Oxford University Press

[B31] Goldberg D Williams P A user’s guide to the General Health Questionnaire 1988 Windsor, UK: NFER- Nelson

[B32] Goldberg D Manual of the general health questionnaire 1978 Windsor, England: NFER Publishing

[B33] Hobbs P Ballinger CB Greenwood C Martin B McLure A Factor analysis and validation of the general health questionnaire in men-a general practice survey Brit J Psychiatry 1984 114 270 275 6704620 10.1192/bjp.144.3.270

[B34] Bellantuono C Fioro R Zanotelli R Tansella M Psychiatric screening in general practice in Italy. A validity study of the GHQ Soc Psychiatry 1987 22 113 117 3589782 10.1007/BF00584015

[B35] Vazquez-Barquero JL Diez Manrique JF Pena C Quintinal RG Labrador Lopez M Two stage design in a community survey Brit J Psychiatry 1986 149 88 97 3779318 10.1192/bjp.149.1.88

[B36] Sen B Wilkinson G Mari J Psychiatric morbidity in primary health care: a two stage screening procedure in developing countries. Cost effectiveness and choice of instruments Brit J Psychiatry 1987 151 33 38 3118997 10.1192/bjp.151.1.33

[B37] Mari JJ Williams P A comparison of the validity of two psychiatric screening questionnaires in Brazil using ROC analysis Psychol Med 1985 15 651 659 4048323 10.1017/s0033291700031500

[B38] Shamasundar C Murthy S Praksb O Prabhakar N Krishma DKS Psychiatric morbidity in a general practice in an Indian city BMJ 1986 292 1713 1715 3089368 10.1136/bmj.292.6537.1713 PMC1340639

[B39] Mari JJ Williams P A validity study of a psychiatric screening questionnaire in primary care in the city of Sao Paulo Brit J Psychiatry 1986 148 23 26 3955316 10.1192/bjp.148.1.23

[B40] Araya R Wynn R Lewis G Comparison of two self administered psychiatric questionnniares (GHQ 12 and SRQ 20) in primary care in Chile Soc Psychiatr Psychiatric Epidemiol 1992 27 68 73 10.1007/BF00789001 1411744

[B41] Goldberg DP Gater R Sartorius N The validity of two versions of GHQ in the Who study of mental illness in general health care Psychol Med 1997 27 191 197 9122299 10.1017/s0033291796004242

[B42] Politi P Piccinelli M Wilkinson G Reliability, validity and factor structure of the 12 item general health questionnaire among young males in Italy Acta Psychiatrica Scand 1994 90 432 437 10.1111/j.1600-0447.1994.tb01620.x 7892776

[B43] Pan P Goldberg DP A comparison of the validity of the GHQ 12 and CHQ 12 in Chinese primary care patients in Manchester Psychol Med 1990 20 931 940 2284399 10.1017/s003329170003662x

[B44] Kuruvilla A Pothen M Philip K Braganza D Joseph A Jacob KS The validation of the Tamil version of the 12 item general health questionnaire Indian J Psychiatry 1999 41 3 217 221 21455393 PMC2962995

[B45] Montazeri A Haririchi AM Shariati M Garmaoudi G Ebadi M Fateh A The 12 item General Health questionnaire (GHQ 12);translation and validation study of the Iranian version Health Qual Life Outcomes 2003 1 66 14614778 10.1186/1477-7525-1-66 PMC280704

[B46] Piccinelli M Bisoffi G Bon M Ciunico L Tansella M Validity and test retest reliability of the Italian version of the 12 item General Health Questionnaire in general practice: a comparison between three scoring methods Comprehensive Psychiatry 1993 34 198 205 8339539 10.1016/0010-440x(93)90048-9

[B47] Gureje O Obikoya B The GHQ 12 as a screening too n a primary care setting Soc Psychiatr Psychiatric Epidemiol 1990 25 276 280 10.1007/BF00788650 2237610

[B48] Chipimo PJ Fylkesnes K Comparative validity of screening instruments for mental distress in Zambia Clin Prac Epidemiol Mental Health 2010 6 4 15 10.2174/1745017901006010004 PMC2858522 20498698

[B49] Bashir K Blizard B Bosanquet A Bosanquet N Mann AH Jenkins R The evaluation of a mental health facilitator in general practice: effects on recognition, management, and outcome of mental illness Brit J Gen Prac 2000 50 626 629 PMC1313772 11042913

[B50] Bedirhan Ustun T Kostanjsek N Rehm J Kennedy C Epping-Jordan J Saxena S Korff MV Pull C in collaboration with WHO/NIH Joint Project Developing the world health organization disability assessment Schedule 2.0 Bull World Health Org 2010 88 815 823 21076562 10.2471/BLT.09.067231 PMC2971503

[B51] Brooks R EuroQol: the current state of play Health Policy 1996 37 53 72 10158943 10.1016/0168-8510(96)00822-6

[B52] Agota Szende and Alan Williams (eds On behalf of The EuroQol Group’s International Task Force on Self-Reported Health) Measuring Self-Reported Population Health: An International Perspective based on EQ-5Dhttp://www.euroqol.org/eq-5d/population-norms.html

[B53] UNESCO http://www.unesco.org/fileadmin/MULTIMEDIA/INSTITUTES/UIL/confintea/pdf/National_Reports/Africa/Africa/Kenya.pdf

[B54] Eldridge SM Ashby D Feder GS Rudnicka AR Ukoumunne OC Lessons for cluster randomized trials in the twenty-first century: a systematic review of trials in primary care Clinical Trials 2004 1 80 90 16281464 10.1191/1740774504cn006rr

[B55] Puffer S Torgerson DJ Watson J Evidence for risk of bias in cluster randomised trials: review of recent trials published in three general medical journals Brit Med J 2003 327 785 789 14525877 10.1136/bmj.327.7418.785 PMC214092

[B56] StataCorp Stata Statistical Software: Release 12 2011 College Station, TEXAS: StataCorp LP

[B57] Kenya National Bureau of Statistics Kenya 2009 Census Datahttp://marsgroupkenya.org/census

[B58] Roland MTD Understanding controlled trials: what are pragmatic trials Brit Med J 1998 316 2859472515

[B59] M H The pragmatic randomised controlled trial Adv Psychiatric Treatment 2002 8 326 333

[B60] Sikorski CLM Konig H Van Den Bussche H Riedel-Heller SG Does GP Training in depression care affect patient outcome? –A systematic review and meta analysis BMC Health Services Res 2012 12 doi:10.1186/1472-6963-1112-1110 10.1186/1472-6963-12-10 PMC3266633 22233833

[B61] Khadivi RSM Gobadi S The efficiency of mental health integration in primary health care: a ten year study Int J Prev Med3 Suppl 2012 1 S139 S145 PMC3399293 22826756

[B62] Araya RRG Fritsch R Gaete J Rojas M Simon G Peters TJ Treating depression in primary care in low income women in Santiago: a randomized controlled trial Lancet 2003 361 995 1000 12660056 10.1016/S0140-6736(03)12825-5

[B63] Chisholm DJS Sekar K Kumar K Murthy S Integration of mental health care into primary care Demonstration cost-outcome study in India and Pakistan Brit J Psychiatry 2000 176 581 588 10974966 10.1192/bjp.176.6.581

[B64] Chisholm DSK Ayuso-Mateos J Saxena S Reducing the global burden of depression. Population level analysis of intervention cost effectiveness in 14 world regions Brit J Psychiatry 2004 184 393 403 15123502 10.1192/bjp.184.5.393

[B65] Jenkins R Othieno C Okeyo S Aruwa J Wallcraft J Jenkins B Exploring the perspectives and experiences of health workers at primary health facilities in Kenya following training Int J Mental Health Syst 2012 7 1 6 10.1186/1752-4458-7-6 PMC3599922 23379737

[B66] Othieno C Jenkins R Okeyo S Aruwa J Wallcraft J Jenkins B Perspectives and concerns of clients at primary health care facilities involved in evaluation of a national mental health training programme for primary care in Kenya Int J Mental Health Syst 2013 7 5 10.1186/1752-4458-7-5 PMC3576266 23343127

[B67] Jenkins R Othieno C Okeyo S Aruwa J Kingora J Jenkins B Health system challenges to integration of mental health delivery in primary care in Kenya-perspectives of primary care health workers BMC Health Serv Res 2013 13 1 368 24079756 10.1186/1472-6963-13-368 PMC3852631

