# Supplementary material for: Short structured general mental health in service training programme in Kenya improves patient health and social outcomes but not detection of mental health problems - a pragmatic cluster randomised controlled trial
Source: Int J Ment Health Syst. 2013 Nov 5;7:25. doi: 10.1186/1752-4458-7-25 (PMC4174904; doi:10.1186/1752-4458-7-25)
Supplement: Additional file 1 — The consort statements for pragmatic trials. [file 1752-4458-7-25-S1.docx]

**Additional file 1.** **The consort statements for pragmatic trials**

**Extension for pragmatic trials-taken from BMJ article:**

**Improving the reporting of pragmatic trials: an extension of the CONSORT statement. BMJ** 2008;337;a2390 *BMJ*

**http://bmj.com/cgi/content/full/337/nov11_2/a2390**

| Heading | Item number | Standard consort description | Extension for pragmatic trial | Page number |
| --- | --- | --- | --- | --- |
| Background | 2 | Scientific background and explanation of rationale | \| Describe the health or health service problem that the intervention is intended to address and other interventions that may commonly \| \| --- \| | 4-5 |
| Participants | 3 | Eligibility criteria for participants; settings and locations where the data were collected | Eligibility criteria should be explicitly framed to show the degree to which they include typical participants and/or, where applicable, typical providers (eg, nurses), institutions (eg, hospitals), communities (or localities eg, towns) and settings of care (eg, different healthcare financing systems) | Eligibility P13  Settings P8-9 |
| Interventions | 4 | Precise details of the interventions intended for each group and how and when they were actually administered | \| Describe extra resources added to (or resources removed from) usual settings in order to implement intervention. Indicate if efforts were made to standardise the intervention or if the intervention and its delivery were allowed to vary between participants, practitioners, or study sites \| \| --- \| \| Describe the comparator in similar detail to the intervention \| | 9, 12-13 |
| Outcomes | 6 | \| Clearly defined primary and secondary outcome measures and, when applicable, any methods used to enhance the quality of measurements (eg, multiple observations, training of assessors) \| \| --- \| | Explain why the chosen outcomes and, when relevant, the length of follow-up are considered important to those who will use the results of the trial | 21 |
| Sample size | 7 | How sample size was determined; explanation of any interim analyses and stopping rules when applicable | If calculated using the smallest difference considered important by the target decision maker audience (the minimally important difference) then report where this difference was obtained | n/a |
| Blinding | 11 | Whether participants, those administering the interventions, and those assessing the outcomes were blinded to group assignment | If blinding was not done, or was not possible, explain why | 14 |
| Participant flow | 13 | Flow of participants through each stage (a diagram is strongly recommended)—specifically, for each group, report the numbers of participants randomly assigned, receiving intended treatment, completing the study protocol, and analysed for the primary outcome; describe deviations from planned study protocol, together with reasons | The number of participants or units approached to take part in the trial, the number which were eligible, and reasons for non-participation should be reported | 17 |
| Generalisability | 21 | Generalisability (external validity) of the trial findings | Describe key aspects of the setting which determined the trial results. Discuss possible differences in other settings where clinical traditions, health service organisation, staffing, or resources may vary from those of the trial | 21-22 |
